# Supplementary material for: The impact of comprehensive geriatric assessment on postoperative outcomes in elderly surgery: A systematic review and meta-analysis
Source: PLoS One. 2024 Aug 28;19(8):e0306308. doi: 10.1371/journal.pone.0306308 (PMC11356442; doi:10.1371/journal.pone.0306308)
Supplement: S2 File — (DOCX) [file pone.0306308.s002.docx]

**search tactics**

#1“Comprehensive geriatric assessment”[Mesh]

#2 Comprehensive geriatric assessment[All Fields] or Comprehensive geriatric assessment [All Fields] or Geriatric evaluation[All Fields] or CGA[All Fields]

#3 #1 or #2

#4 “Elder patient”[Mesh]

#5 Geriatric* [All Fields] or Psychogeriatric* [All Fields] or Psycho-geriatric* [All Fields] or Gerontolog* [All Fields] or Elder* [All Fields] or Older adult* [All Fields] or Older person* [All Fields] or Older people [All Fields] or Older population* [All Fields] or Old-age or Senior* [All Fields]

#6 #4 or #5

#7 #3 and #6

#8 "Surgery"[Mesh]

#9 Surgery[All Fields] or Operative [All Fields] or Procedure [All Fields] or Surgical [All Fields]

#10 #8 or #9

#11 #7 and #10

#12"Length of Stay"[Mesh]

#13 Length of Stay[All Fields] or Stay Length[All Fields] or Stay Lengths[All Fields] or Hospital Stay[All Fields] or Hospital Stays[All Fields] or Hospitalization[All Fields]

#14"Frailty"[Mesh]

#15 Frailty[All Fields] or Frailties[All Fields] or Frailness[All Fields] or Frailty Syndrome[All Fields] or Debility[All Fields] or Debilities[All Fields] or Asthenia[All Fields] or Weakness[All Fields]

#16"Delirium"[Mesh]

#17 Delirium[All Fields] or Delirious Speech[All Fields] or Deliration[All Fields] or Deliriousness[All Fields] or Phrenitis[All Fields]

#18"Infections"[Mesh]

#19”30 Day Readmission“[Mesh]

#20 30 Day Readmission*[All Fields] or Thirty Day Readmission*[All Fields] or 30-days re-admission*[All Fields]

#21"Adverse effects"[Mesh]

#22 Adverse effects[All Fields] or Adverse outcomes[All Fields] or Patient Outcome[All Fields] or Postoperative Complications[All Fields] or Postoperation[All Fields] or Postoperative outcomes[All Fields]

#23 #12 or #13 or #14 or #15 or #16 or #17 or #18 or #19 or #20 or #21 or #22 and #11

#24 randomized controlled trial [pt] OR controlled clinical trial [pt] OR randomized [tiab] OR placebo [tiab] OR clinical trials as topic [mesh: no exp] OR randomly [tiab] OR trial [ti] NOT (animals [mh] NOT humans [mh])

#25 #23 and #24
